# Keratinocyte EGF signalling dominates in atopic dermatitis lesions: A comparative RNAseq analysis

**DOI:** 10.1111/exd.14605

**Published:** 2022-05-15

**Authors:** Kate Timms, Hui Guo, Peter Arkwright, Joanne Pennock

**Affiliations:** ^1^ Lydia Becker Institute of Immunology and Inflammation, School of Biological Sciences, Faculty of Biology Medicine and Health University of Manchester Manchester UK; ^2^ Center for Biostatistics, School of Health Sciences, Faculty of Biology Medicine and Health The University of Manchester Manchester UK; ^3^ Department of Paediatric Allergy & Immunology Royal Manchester Children's Hospital Manchester UK

**Keywords:** eczema, immunopathology, inflammation, skin, Th2

## Abstract

Atopic dermatitis (AD) remains a highly heterogenous disorder with a multifactorial aetiology. Whilst keratinocytes are known to play a fundamental role in AD, their contribution to the overall immune landscape in moderate‐to‐severe AD is still poorly understood. In order to design new therapeutics, further investigation is needed into common disease pathways at the molecular level. We used publicly available whole‐tissue RNAseq data (4 studies) and single‐cell RNAseq keratinocyte data to identify genes/pathways that are involved in keratinocyte responses in AD and after dupilumab treatment. Transcripts present in both keratinocytes (single‐cell) and whole‐tissue, referred to as the keratinocyte‐enriched lesional skin (KELS) genes, were analysed using functional/pathway analysis. Following statistical testing, 2049 genes (16.8%) were differentially expressed in KELS. Enrichment analyses predicted increases in not only type‐1/type‐2 immune signalling and chemoattraction, but also in EGF‐dominated growth factor signalling. We identified complex crosstalk between keratinocytes and immune cells involving a dominant EGF family signature which converges on keratinocytes with potential immunomodulatory and chemotaxis‐promoting consequences. Although keratinocytes express the IL4R, we observed no change in EGF signalling in KELS after three‐month treatment with dupilumab, indicating that this pathway is not modulated by dupilumab immunotherapy. EGF family signalling is significantly dysregulated in AD lesions but is not associated with keratinocyte proliferation. EGF signalling pathways in AD require further study.

## Graphical abstract

We have used RNAseq comparative analysis to demonstrate specific crosstalk between immune cells and lesional keratinocytes (KELS) from atopic dermatitis patients. We show for the first time that the KELS transcriptome is dominated by EGF family receptors and downstream signalling alongside the response to type‐1 and type‐2 cytokines.

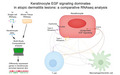

**Abbreviations**
AD: atopic dermatitis
AREG: amphiregulin
DEG: differently expressed gene
EGF: epidermal growth factor
EGFR: epidermal growth factor receptor
ERBB2: Erb‐B2 Receptor Tyrosine Kinase 2
ERBB4: Erb‐B2 Receptor Tyrosine Kinase 4
EREG: epiregulin
HBEGF: heparin‐binding epidermal growth factor
*IFNG*
: interferon gamma (gene)
IFNγ: interferon gamma (protein)
*IL33*
: interleukin 33 (gene)
IL‐33: interleukin 33 (protein)
*IL4*
: interleukin 4 (gene)
IL‐4: interleukin 4 (protein)
*IL4R*
: interleukin 4 receptor alpha (gene)
IL4RA: interleukin 4 receptor alpha (protein)
IPA: ingenuity pathway analysis
JAK: Janus kinase
KELS: keratinocyte‐enriched lesional skin
NRG1: neuregulin 1
PDGF: platelet‐derived growth factor
PlGF: placental growth factor
RNAseq: RNA sequencing
TGFA: transforming growth factor alpha
Th1: T helper 1 cells
Th17: T helper 17 cells
Th2: T helper 2 cells
TNF: tumour necrosis factor alpha (gene)
TNFα: tumour necrosis factor alpha (protein)
TSLP: thymic stromal lymphopoietin
TYK2: tyrosine kinase 2
VEGF: vascular endothelial growth factor
VEGFA: vascular endothelial growth factor A

## 1 INTRODUCTION

Atopic dermatitis (AD) is a chronic inflammatory skin disorder affecting 20% of children and 2% of adults in Europe.^1^, ^2^ AD is classically described as a type‐2 driven disease with increased circulating IL‐33 and thymic stromal lymphopoietin (TSLP)^3^ alongside a lack of inflammasome activation in lesional skin.^4^, ^5^ Whilst genome‐wide association studies have identified 31 AD susceptibility loci^6^ (e.g. barrier protein filaggrin^7^), these only account for 30% of AD heritability.^6^, ^8^ Environmental factors such as the skin microbiome are also important, as *Staphylococcus aureus* (*S. aureus*) colonization is increased in AD patients,^9^ often preceding symptom onset.^10^ This multi‐faceted aetiology of AD is reflected in the heterogeneity of clinical presentation^11^ and lesional transcriptome.^12^

The need for more effective therapy for AD is partly met by biologics for the treatment of moderate‐to‐severe AD.^13^, ^14^, ^15^ Dupilumab is a monoclonal antibody directed towards the IL‐4 receptor alpha, an integral component of the IL‐4 and IL‐13 receptors which drive Th2 immune responses. The clinical success of dupilumab supports a mechanistic role for type‐2 pathways in AD, demonstrating that there are inflammatory genes/pathways in common amongst AD patients.

Keratinocytes are master producers of the classic type‐2 AD cytokines IL‐33 and TSLP,^16^, ^17^ suggesting a role for these cytokines upstream of observed immune pathology. However, little is known of the in situ keratinocyte transcriptome during AD.^18^ There are only a handful of published tissue‐level RNAseq studies in AD patients which have prioritized analysis of the adaptive immune response. Here, we investigate whether the transcriptomic changes seen in keratinocytes, as identified by the only keratinocyte single‐cell sequencing study,^19^ are reproduced in whole‐tissue RNAseq, and whether they provide clues to additional cell signalling pathways which may modulate the skin inflammatory response. We use data mining of publicly available RNAseq studies to undertake this keratinocyte‐enriched transcriptomics approach, to identify novel keratinocyte‐centred pathways integral to lesional AD. This approach to identification of common transcriptomic signatures may aid the development of novel therapeutics for AD, upstream of the currently available immunotherapy.

## 2 METHODS

### 2.1 Search strategy and data acquisition

The National Centre for Biotechnology Information Gene Expression Omnibus database^20^, ^21^ was used to identify suitable datasets. Figure S1 outlines the pipeline used to identify studies. The five suitable studies were:^12^, ^19^, ^22^, ^23^, ^24^ All studies were conducted on adult moderate‐to‐severe AD patients. Full patient and study demographics are given in Table 1. All data were imported as TXT files into R 4.0.2.

**TABLE 1:** Demographics and skin scores of patients used in selected studies

Study	He et al., 2020	Tsoi et al., 2019	Suárez‐Fariñas et al., 2015	Mack et al., 2020	Möbus et al., 2020
Accession [Reference]	GSE147424 [49]	GSE121212 [50]	GSE65832 [51]	GSE140227 [52]	GSE157194 [53]
Sex	3 Male	17 Male	12 Male	3 Male	41 Male
	2 Female	10 Female	8 Female	3 Female	16 Female
Age (years)	36.8 ± 11.6	34.1 ± 11.0	45.6 ± 13.7	44.8 ± 8.4	43.6 ± 15.3
SCORAD or EASI	SCORAD 68.7	SCORAD 31.1 ± 11.0	SCORAD 63.3 ± 14.0	EASI 34.6 ± 4.8	EASI 20.2 ± 11.7
RNA‐seq Platform	Illumina HiSeq2500	Illumina HiSeq2500	Illumina HiSeq2500	Illumina HiSeq3000	Illumina HiSeq3000

*Note:* Data are mean ± standard deviation.

### 2.2 Data and statistical analysis

As shown in Figure S1, data from each of the five RNAseq studies were integrated, with only those genes identified in all studies being included in analysis. Transcript variants were not available for all studies and so are not discussed here. The Bioconductor suite and ggplot2, ggpubr, biomaRt, grid, gridExtra, tidyverse, pheatmap, Clustvis, corrplot and matrixTests packages were used for data analyses and presentation.

To allow for the enrichment of keratinocyte‐expressed genes in whole‐tissue, keratinocyte single‐cell data^19^ were used. 276 genes were excluded as they were detected in only one patient^19^ (Table S1). As expected for single‐cell sequencing, the read number for transcripts was substantially lower than that for the whole‐tissue analyses, rendering batch correction inappropriate. Participant matching of lesional and non‐lesional samples was not always available, precluding the use of normalized reads without bias. Therefore, lesional or non‐lesional gene expression was averaged for every gene in each study, followed by calculation of log2 fold change between lesional and non‐lesional skin. These data were used for all further comparisons.

Normality at the gene level was calculated using the Shapiro–Wilk test (*n* = 5; 72.3% normally distributed; *p* > 0.05). Non‐normally distributed genes (3376) were excluded. For the normally distributed genes, a one sample t‐test was performed on the log2 fold change values for each gene to identify deviation from 0 (i.e. no change between lesional and non‐lesional skin), followed by *p*‐value correction for false discovery rate using the Benjamini and Hochberg (BH) method. Reported *p*‐values are the adjusted values (*q*‐values; Table S2). To compare data from the current study with microarray data from keratinocytes treated with TNFα,^25^ IL‐1β,^26^ IFNα,^27^ IFNγ,^26^, ^27^, ^28^ IL‐4^27^, ^29^ and IL‐13,^27^ and RNAseq data from skin from dupilumab‐treated patients,^24^ Pearson correlations were used. For comparisons between cell types,^19^ a Kruskal–Wallis test with Dunn's post hoc test was used. For comparisons between lesional and non‐lesional skin between cell types, a mixed‐effects model with multiple comparisons was used, adjusted for using the BH method.

### 2.3 Functional, pathway and upstream regulator analysis

All functional, pathway and upstream regulator enrichment analyses were performed using ingenuity pathway analysis (IPA) (Summer 2020 Release; Qiagen Inc., https://www.qiagenbioinformatics.com/products/ingenuitypathway‐analysis).^30^ Fisher's exact tests identified enrichment, followed by BH multiple testing correction (*q*‐values are reported). Functions/pathways relating to non‐keratinocyte cell types were excluded. GraphPad Prism 8 was used for additional data presentation. Z‐scores reported in this study refer to predicted molecule/pathway/function activity, with positive z‐scores indicating upregulation and negative z‐scores indicating downregulation. To be considered significant, a z‐score must be >2 or <−2, though those which do not meet this threshold are reported for completeness.

## 3 RESULTS

### 3.1 Comparison of atopic dermatitis RNAseq datasets

The recently published keratinocyte single‐cell transcriptome was compared to whole‐tissue AD RNAseq datasets.^12^, ^19^, ^22^, ^23^, ^24^ A total of 12 174 common genes were identified across age and gender‐matched adult patients (Table S1). Of these, 2049 genes (16.8%) were significantly up‐ or downregulated across all datasets (Table S2).

The keratinocytes identified by He et al.^19^ showed a greater degree of variation in log2fold gene expression than did the whole‐tissue RNAseq studies. This is expected, as tissue level RNAseq “dilutes” larger expression fluctuations seen in individual cell populations. However, in order to ensure that inflammatory signals detected in the keratinocytes were not derived from contaminating immune cells, we compared markers for keratinocytes, T cells and macrophages/dendritic cells across their respective cell clusters (Figure S1A‐C) as several other cell types were present in the single‐cell study.^19^ Expression of cell‐specific markers was restricted/enriched to the appropriate cell type(s) and whole transcriptomes differed (Figure S1D), confirming the integrity of the keratinocyte dataset.

Single cell and whole‐tissue datasets were significantly positively correlated with one another (*p* < 0.0001; Figure S1B), particularly when comparing DEGs (Figure S1C). This validates the comparative methodology, suggesting that keratinocyte‐expressed DEGs identified across datasets are meaningful for keratinocytes. The term keratinocyte‐enriched lesional skin (KELS) is therefore used to describe the multi‐study lesional data in our analysis.

### 3.2 Functional analysis of differently expressed genes in keratinocyte‐enriched lesional skin of atopic dermatitis patients

**FIGURE 1:**
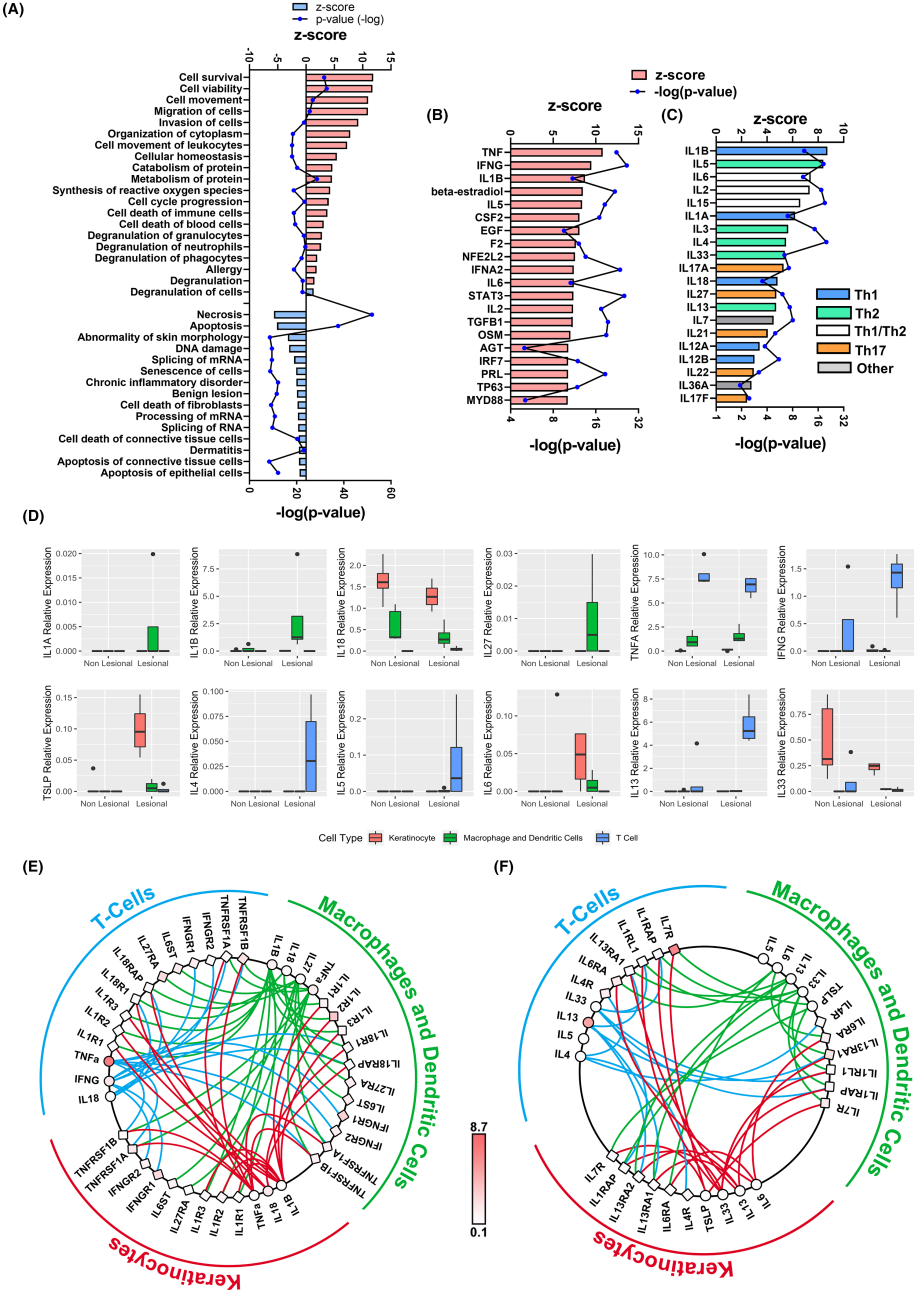
Enrichment analysis of keratinocyte‐enriched lesional skin predicts increased type‐1 and type‐2 inflammation. (A) Most significantly enriched functions predicted to be activated (red) or inactivated (blue) by differently expressed genes (DEGs) between keratinocyte‐enriched lesional (KELS) and non‐lesional skin. IPA activity z‐score denotes activation. Upregulated functions are grouped in the pie chart. All regulators (B) or interleukins (C) predicted to be increased (red) or decreased (blue) upstream of the KELS DEGs. (D) The expression of key type‐1 (top) and type‐2 (bottom) cytokines in in situ keratinocytes, T cells and macrophages/dendritic cells in atopic dermatitis skin. Crosstalk between (E) type‐1 and (F) type‐2 ligands (circles) and receptors (squares) between in situ keratinocytes, T cells and macrophages/dendritic cells in atopic dermatitis lesional skin (coloration is relative lesional expression). DEGs are considered *p* < 0.05, **p < 0.01, ****p < 0.0001

Next, we examined the impact of KELS DEGs using functional enrichment analysis. We identified that KELS DEGs show signatures of allergy and are involved in immune cell chemotaxis and activation (Figure 1A). Additionally, AD lesional keratinocytes upregulate genes key to the expansion of connective tissue, cell survival and viability, likely reflecting AD epidermal hyperplasia.^31^ Amongst the top‐ranked inactivated functions (z‐score < 0) are cell death‐associated functions (i.e. apoptosis, necrosis and DNA damage; Figure 1A). The function designated as “dermatitis” also has decreased activity in KELS, suggesting that AD may be distinct from other dermatoses on which this classification is based. These data support published literature demonstrating that chemotaxis of leukocytes and maintenance of cell viability are key elements of the keratinocyte‐driven immune pathology during AD.^32^, ^33^

### 3.3 Type‐1‐ and Type‐2‐related inflammatory processes co‐exist in keratinocyte‐enriched lesional skin of atopic dermatitis patients

It is possible to statistically identify “upstream regulators” which have been experimentally shown to cause DEGs. These regulators are predicted to be upstream, and therefore potentially causative of observed gene expression changes. As expected, specific DEGs in KELS were associated with a predicted increase in several upstream pro‐inflammatory mediators including Th2‐, Th1‐ and Th17‐associated factors (e.g. *TNF*, *IFNG*, *IL1B* and *IL6*; Figure 1B and C; Table S4) highlighting the interplay between keratinocytes and immune cells in moderate‐to‐severe AD (Figure 1C). This is further evidenced by specific inflammatory and chemotactic signalling pathways enriched in KELS DEGS (Figure S1A‐B). Though both Th1 and Th2 profiles in AD lesional skin have been well documented,^34^ our analysis highlights both their predicted impact on lesional keratinocytes and their concurrence.

To confirm this upstream pathway analysis, DEGs from the current study were compared to published data from keratinocytes treated in vitro with IFNγ, IFNα, TNFα, IL‐1β, IL‐4 or IL‐13.^25^, ^26^, ^27^, ^28^, ^29^ Positive correlations were seen between KELS DEGs and keratinocytes treated with Th1‐related cytokines (IFNγ, TNF‐α and IL‐1β) and the Th2 cytokine IL‐4 (Figure S1A). These data are supported by the higher *IL4R* expression in lesional keratinocytes compared with non‐lesional keratinocytes (Figure S1B and C). However, treatment of keratinocytes with IL‐13 resulted in a weak negative correlation, perhaps due to significant lesional keratinocyte upregulation of the non‐functional IL‐13 decoy receptor *IL13RA2* (Figure S1C), which is overexpressed in lichenified lesional AD skin.^35^ All KELS inflammatory DEGs are shown in Figure S1.

In order to gain a deeper understanding of the inflammatory drivers of KELS, we next compared expression of key type‐1 and type‐2 immune transcripts across keratinocyte, T cell and macrophage/dendritic cell single‐cell profiles (Figure 1D).^19^ We identified that T cells are the primary source of *IFNG* (IFNγ), *TNF* (TNFα), *IL4* (IL‐4) and *IL13* (IL‐13), macrophages/dendritic cells are the main producer of *IL1B* (IL‐1β) and keratinocytes are significant producers of *TSLP*, *IL6* and *IL33* in AD lesional skin. To further clarify the role of keratinocytes in immune‐crosstalk, we mapped key type‐1 (Figure 1E) and type‐2 (Figure 1F) ligand–receptor interactions. Type‐2 cytokines *IL6*, *IL13*, *IL33* and *TSLP* along with their receptor transcripts were all expressed by keratinocytes. *IL4R* (IL4RA) has the highest expression of any type‐2 receptor in keratinocytes. Whilst all type‐1 cytokines, excepting *IL18*, are expressed at higher levels in T cells or macrophages/dendritic cells, keratinocytes did transcribe measurable quantities of *IL1B*, *IL18* and *TNF*. The striking interconnectivity of the cytokine signalling pathways between keratinocytes and immune cells shows the central and specific role that keratinocytes play in the immunopathology of AD lesional skin.

### 3.4 Growth factor and hormone transcripts in lesional keratinocytes

**FIGURE 2:**
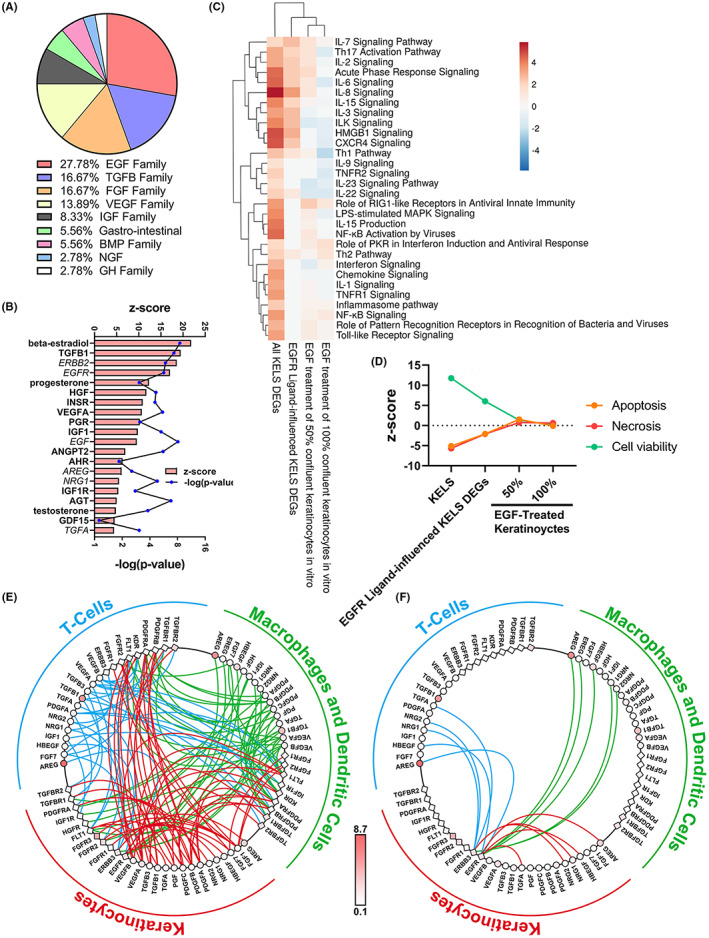
Growth factor signalling is altered in atopic dermatitis lesional skin. (A‐B) Growth factor upstream mediators grouped by family in (A) and the most significantly enriched growth factors and hormones given in (B). (C) Inflammatory pathways enriched in differently expressed genes (DEGs) between keratinocyte‐enriched lesional (KELS), KELS DEGs downstream of EGFR ligands and non‐lesional skin and in EGF‐treated human keratinocytes.^36^ (D) Cell viability/death pathways in KELS, KELS DEGs downstream of EGFR ligands and in EGF‐treated human keratinocytes.^36^ (E,F) Ligand–receptor interaction plots between in situ keratinocytes, T cells and macrophages/dendritic cells for all (E) growth factors or (F) EGF family ligands in AD lesional skin. Ligands (circles) and receptors (squares) are coloured by relative expression in lesional skin. DEGs are considered *p* < 0.05 using a one‐sample t‐test with multiple testing correction using the BH method. Heatmaps use Euclidean distance and complete linkage

Growth factor pathways and regulators, particularly the epidermal growth factor (EGF) family, featured significantly in our KELS DEGs analysis (Figure 2A‐B). EGF family ligands (neuregulin 1 (NRG1), amphiregulin (AREG; 2.1‐fold upregulated in KELS, *p* < 0.05), epiregulin (EREG), transforming growth factor alpha (TGFA), heparin‐binding EGF (HBEGF; 2.3‐fold upregulated in KELS; *p* < 0.05), EGF) and receptors (Erb‐B2 Receptor Tyrosine Kinase 2 [ERBB2], ERBB4 and EGFR) were predicted as significant upstream regulators of KELS DEGs (Figure 2B; Table S4; Figure S1A).

To support these data, DEGs from the current study were compared with EGF‐treated post‐confluence keratinocytes,^36^ with which they were significantly if not strongly positively correlated (Figure S1C‐D). This suggests that EGF has the potential to stimulate at least some of the gene expression changes seen in AD lesional keratinocytes. Cell viability/death pathway activity was not activated/inactivated in EGF‐treated keratinocytes (Figure 2D, z‐score <2). Pathway enrichment analysis identified that EGF‐treated keratinocytes share several similarly altered inflammatory pathways with our KELS DEGs (Figure 2C). Th2, interferon, NFκB, inflammasome, IL‐7, toll‐like receptor and pattern recognition receptor signalling were all increased in KELS and EGF‐treated keratinocytes. Th1 and Th17 signalling, along with a number of other inflammatory pathways were suppressed by EGF treatment of keratinocytes (Figure 2C).

Following the confirmation that EGF can have pro‐inflammatory effects in keratinocytes, we identified KELS DEGs for which EGFR ligands were upstream regulators to bioinformatically predicted the impact of EGFR ligand signalling in situ in patient KELS. Inflammatory pathways from this subset of DEGs cluster with EGF‐treated keratinocytes in vitro (Figure 2C) evidencing the validity of this approach. Of note, whilst NFκB signalling, interferon signalling and the inflammasome pathway are all increased in KELS and in vitro EGFR treated keratinocytes, these pathways are not predicted to be altered by EGFR signalling patient lesional keratinocytes (Figure 2C). However, part of the activation of Th1 (z‐score 1.34) and Th2 (z‐score 1) pathways and IL15 (z‐score 2), IL‐22 (z‐score 1), IL‐3 (z‐score 1.65), IL‐6 (z‐score 2.34), IL‐7 (z‐score 1.65), IL‐8 (z‐score 3.46) and CXCR4 (z‐score 2.50) signalling appear to be driven by EGFR signalling in AD lesional keratinocytes (Figure 2C). Whilst many of these pathways do not reach significant upregulation (z‐score ≥2.0 cut‐off) downstream of EGFR ligands, they appear to account for part of the ≥2.0 z‐score activation of these pathways in KELS overall. Whilst DEG downstream of EGFR ligands were not predicted to influence activity of wound healing in KELS, part of the increase in cell viability (z‐score 6.01 vs 11.74 in all DEGs) and decrease in apoptosis (z‐score − 2.75 vs. −3.82 in all DEGs) appears to be downstream of EGFR signalling (Figure 2C). There was no influence on keratinocyte or general cell proliferation downstream of EGFR ligands.

Vascular endothelial growth factor (VEGF) family ligands *VEGFA* (2.0‐fold upregulated in KELS; *p* < 0.01) and *PlGF* (3.6‐fold upregulated in KELS; *p* < 0.05) are also predicted to lie upstream of KELS DEGs and are significantly upregulated in KELS, suggesting active VEGF signalling in keratinocytes. A summary of growth factor transcripts altered in KELS are given in Figure S1B. Alongside growth factors, several steroid hormones are predicted to have contribute to KELS DEGs, particularly estrogens (Figure 2B). However, further analysis of sex hormones was prevented by the inability to track their levels by transcriptomics and a lack of complete patient sex information, precluding stratification by sex.

Keratinocytes, T cells and macrophages/dendritic cells share complex growth factor crosstalk (Figure 2E‐F). All three cell types produce EGFR ligands (Figure 2F), but only keratinocytes express EGFR and therefore have the potential to respond in both an autocrine and paracrine manner. PDGF/VEGF crosstalk also occurs (Figure S1). Taken together, these data suggest that both EGF and VEGFA pathways are altered in lesional keratinocytes.

As such, these data suggest that signalling through the EGFR appears to have some pro‐inflammatory and pro‐cell survival consequences in situ in AD patient KELS.

### 3.5 The impact of dupilumab on inflammatory and growth factor pathways in lesional skin

In targeting the IL4RA, dupilumab blocks both the IL‐4 and IL‐13 pathways, inhibiting type‐2 responses with greater efficacy than inhibition of IL‐13 alone.^37^ Whilst dupilumab improves barrier function, epidermal hyperplasia and expression of type‐2‐related cytokines,^31^ the effects of dupilumab on the keratinocyte transcriptome are unknown. We therefore analysed the effect of 3 months dupilumab treatment^24^ on KELS.

**FIGURE 3:**
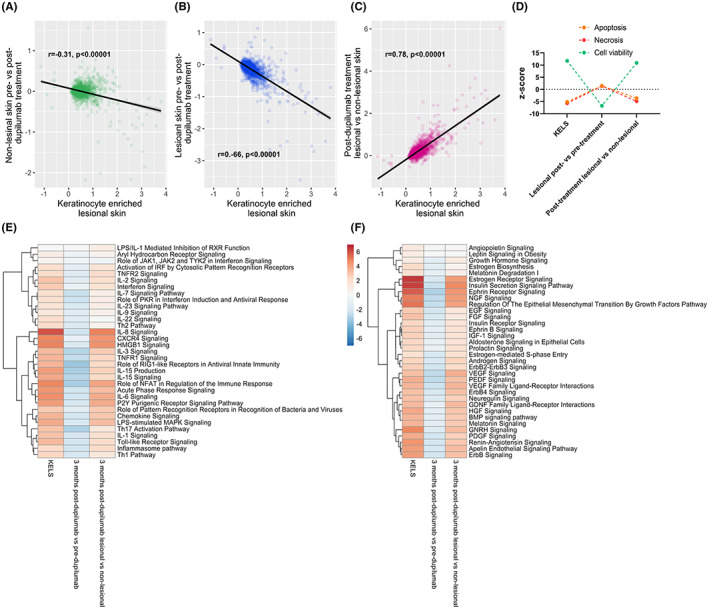
Dupilumab impacts on inflammatory and growth factor and hormone pathways associated with keratinocyte‐enriched lesional skin in atopic dermatitis. (A–B) Publicly available RNAseq data from the lesional skin of atopic dermatitis patients treated with dupilumab^24^ were correlated with keratinocyte‐enriched lesional skin (KELS) differently altered genes (DEGs) from the current study. (C) The impact of dupilumab treatment on cell viability and apoptosis. (D) Inflammatory pathways enriched in skin from dupilumab treated patients which were also enriched in the KELS DEGs from the current study. (E) As in D, but for growth factor and hormone pathways

As predicted, RNAseq from both keratinocyte‐enriched non‐lesional and lesional skin of patients treated with dupilumab for 3 months (log2 foldchange from pre‐treatment) demonstrated a significant reduction in the aberrant transcription post‐treatment (Figure 3A‐B). However, the strength of the negative correlation between non‐lesional skin post‐dupilumab treatment and KELS is less than that between lesional skin and KELS, which may reflect the greater magnitude of aberrant gene expression in untreated lesional skin. Additionally, as IL4R was increased 2.1‐fold in lesional skin in our current study and 1.7‐fold in the keratinocyte single‐cell analysis,^19^ we predict that at least part of the greater magnitude of treatment on lesional keratinocytes is via higher levels of IL4R.

Given the impact of dupilumab on non‐lesional skin, it is reasonable to assume that dupilumab may be having a systemic impact on skin. To analyse the specific impact on lesional keratinocytes (i.e. how well dupilumab is normalizing lesional skin to a non‐lesional transcriptome), we next compared post‐dupilumab lesional vs post‐dupilumab non‐lesional skin to KELS DEGs. We believe this intra‐patient control gives the most accurate picture of the lesional transcriptome as it controls for systemic impacts which may otherwise hide remaining defects in lesional skin. We found that KELS DEGs were still significantly positively correlated with post‐dupilumab lesional versus non‐lesional skin, suggesting that keratinocyte signature AD‐related gene expression changes remain in lesional skin post‐treatment (Figure 3C). As such, it appears that the majority of the improvement in lesional skin transcriptome is due to the systemic effects of dupilumab, meaning that the deficit between lesional and non‐lesional skin remains. In agreement with previous studies,^31^ our pathway enrichment analysis predicted that dupilumab decreases type‐2 pathway signalling, as well as type‐1 and associated pathways in lesional skin compared to pre‐treatment, but not when normalized to non‐lesional skin post‐treatment (Figure 3E). Although cell death was increased and viability decreased in lesional skin post‐dupilumab treatment, this was not the case when compared with non‐lesional skin in treated patients (Figure 3D).

Growth factor signalling, including EGF and VEGF/PDGF pathways, and steroid hormone signalling also remained elevated in post‐dupilumab lesional skin versus post‐dupilumab non‐lesional skin (Figure 3F). Given this sustained elevation, we compared just those DEGs known to be downstream of EGFR ligands. There was no significant difference (i.e. >2 difference in z‐score) observed in inflammatory pathways nor in cell survival or apoptosis following dupilumab treatment (data not shown). Overall, these data suggest that after 3 months of dupilumab treatment, the lesional skin transcriptome is still significantly different to non‐lesional skin, showing ongoing inflammatory and growth factor signalling.

### 3.6 Dupliumab selectively impacts chemotaxis in lesional skin

**FIGURE 4:**
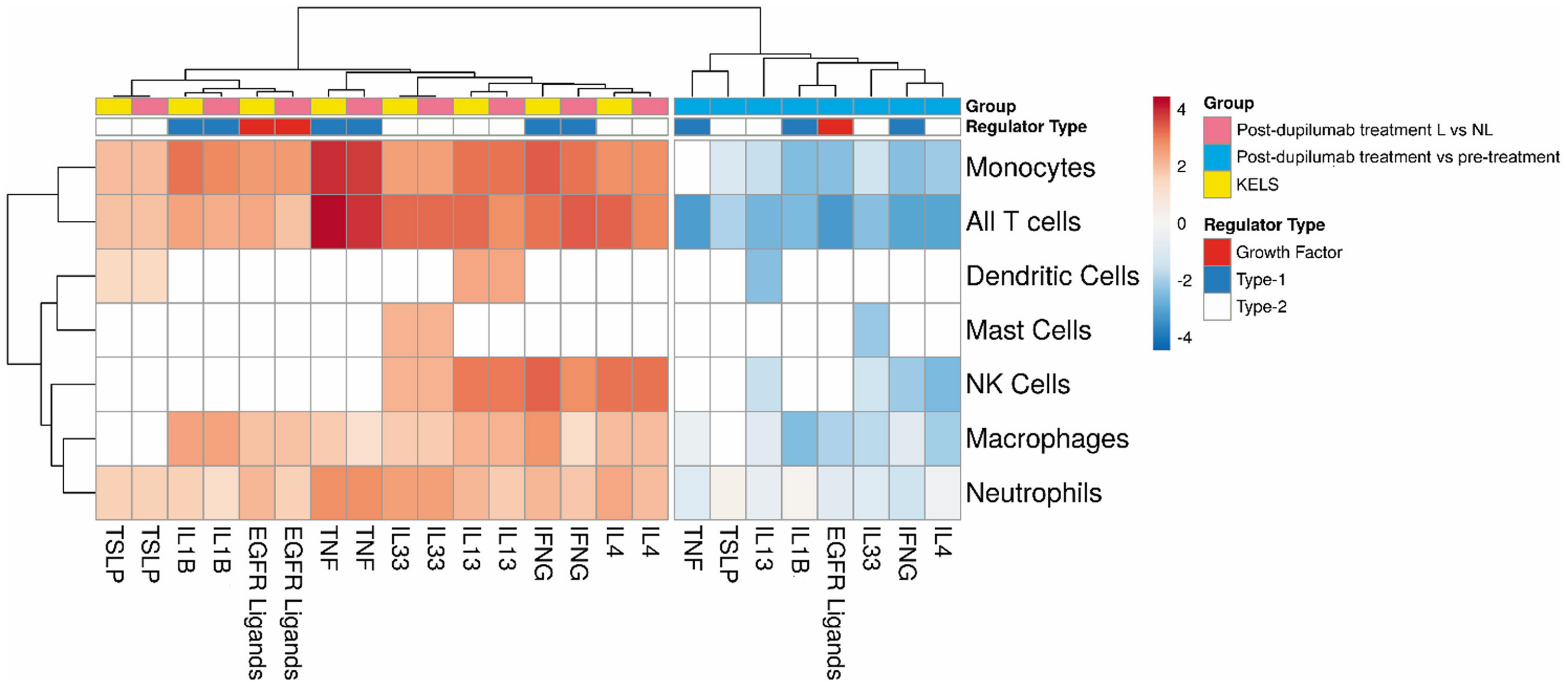
Regulation of keratinocyte‐induced chemotaxis of immune cells by upstream inflammatory and EGFR signalling. Regulators predicted to be upstream of keratinocyte‐enriched lesional skin differently expressed genes (KELS DEGs) were identified using ingenuity pathway analysis (IPA). The KELS DEGs responsible for chemotaxis and known to be downstream of these regulators were then analysed using IPA, with the predicted promotion or chemotaxis of individual immune cell types given by the activity z‐score. Cell types and upstream regulators were clustered using Euclidean distance and complete linkage. Cluster 1: Post‐dupilumab treated lesional versus non‐lesional skin, KELS; Cluster 2: Post‐dupilumab treatment versus pre‐treatment lesional skin. White coloration represents zero significant change

One of the major roles of keratinocytes in AD is chemotaxis of immune cells, which we replicated in our functional enrichment analysis (Figure 1A, Figure S1B). IFNγ, TNFα, IL‐1B, IL‐4, IL‐13, IL‐33 and EGFR ligand‐regulated KELS DEGs lie upstream of chemotactic factors driving infiltration of specific immune cells (Figure 4). Given the expression of EGFR by keratinocytes and the production of EGFR ligands by T cells and macrophages/dendritic cells (Figure 2E‐F), this interaction could drive positive feedback, perpetuating cell infiltration through expression of chemotactic factors, and lesional inflammation. In post‐dupilumab treated lesional versus non‐lesional skin, the transcription of chemotactic factors did not differ from KELS (Figure 4, Cluster 1), demonstrating that this short treatment window likely does not significantly resolve lesional immune cell infiltration. Some changes can be seen in pre‐ versus post‐dupilumab treatment of lesional skin alone (Figure 4, Cluster 2), but dupilumab has no impact on EGFR ligand driven expression of factors known to chemoattract NK cells, mast cells and dendritic cells.

## 4 DISCUSSION

Impaired epidermal barrier function leads to increased environmental antigen exposure, allowing keratinocytes to drive Th2 immune responses through the release of IL33 and TSLP leading to AD. The clinical success of dupilumab demonstrates that inhibition of the downstream Th2 immune response can reduce immunopathology, but whether this can achieve disease resolution remains to be seen. Here, we use transcriptome comparison across five studies to expand our understanding of keratinocyte cellular responses and their role in moderate‐to‐severe AD. Alongside the expected inflammatory signals, we clearly show a dysregulation of growth factor pathways with EGF family signalling dominating. As expected, cytokines from innate and adaptive immune cells (e.g. *IFNG, TNF, IL1B, IL33* and *IL4*) were predicted to be upstream of differentially expressed genes in KELS, demonstrating the temporal co‐existence of type‐1 and type‐2 immune responses at the transcriptional level, perpetuating inflammation and driving chemotaxis. However, both keratinocytes and immune cells are involved in a complex crosstalk of cytokines, receptors and growth factors. Although 3 months of dupilumab therapy reduces the inflammatory transcriptome in treated AD skin, there remains significant upregulation of both immune and EGF family signalling in lesional keratinocytes.

The EGF signalling network is one of the most complex known.^38^ EGF family members are overexpressed during wound healing and psoriasis,^38^ but their importance in atopic dermatitis is unclear.^39^, ^40^ In mouse models, EGFR signalling ameliorates AD,^39^, ^41^, ^42^ but levels of EGFR and its ligands in AD lesions are debated.^39^, ^40^ Here, we show that EGF family signalling dominates growth factor pathways in KELS. EGFR ligands HBEGF and AREG are upregulated in KELS, and KELS DEGs are correlated with and share enriched upregulated inflammatory pathways with EGF‐treated in vitro keratinocytes, suggesting autocrine signalling could occur in these cells. As AD lesional bone‐marrow‐derived immune cells express several EGFR ligands but not EGFR, it is likely they promote EGFR signalling in AD keratinocytes. It is possible, therefore, that the promotion of chemotactic factors for these cells downstream of EGFR ligands in keratinocytes identified in this study may represent a positive feedback loop, perpetuating infiltration of immune cells and thus pro‐inflammaotry EGFR signalling. Whilst downstream consequences of EGFR signalling include proliferation/survival of keratinocytes, promoting re‐epithelialization and cutaneous healing,^38^ our analysis showed no significant alteration in proliferation or repair in KELS, suggesting an alternative EGFR signalling outcome in AD lesions. Instead, EGFR signalling appears to contribute to maintaining cell viability and promoting inflammation through various pathways.

It has been proposed that the local inflammatory environment directs the consequences of EGFR signalling in keratinocytes. For example, EGFR activation by *Staphylococcus aureus* ^43^ or house dust mite exposure^44^ promotes keratinocyte IL‐1α / IL‐1β or IL‐33 production, respectively. EGFR activation by HB‐EGF prevents *Staphylococcus aureus* infection in human skin explants, possibly by increasing beta‐defensin‐3 secretion.^45^, ^46^ Increased EGF signalling may explain dupilumab‐induced reduced *Staphylococcus aureus* colonization^47^ and other infections,^48^ potentially by increasing filaggrin and loricrin expression.^41^ Given the prevalence of *Staphylococcus aureus* colonization in AD,^9^ EGFR activation may in fact contribute to the healing response in AD, helping to restore epithelial barrier function and reduce bacterial colonization.

It is has been demonstrated by several independent studies that dupilumab drives marked clinical improvement in AD over relatively short timescales.^13^, ^14^, ^15^, ^31^ Our data demonstrate that 12 weeks of dupilumab treatment leads to overall reduced abnormal keratinocyte transcription, whilst ongoing inflammatory responses remain in the lesional skin cells of these patients.^31^ This may be a result of continued exposure to environmental triggers, as clinical observations report marked but not complete improvement in disease.^31^ Whilst dupilumab is associated with decreased *Staphylococcus aureus* number on lesional skin during treatment, the bacteria do re‐colonize after cessation of treatment.^47^ Additionally, the inability of dupilumab to normalize EGF family signalling, which we have shown to be pro‐inflammatory and pro‐cell viability in lesional keratinocytes, suggests that this maintained EGF signalling could be a contributing factor to ongoing lesional inflammation post‐treatment.

We acknowledge several limitations of the current data and, thus, our analyses. Only one single‐cell study was available, precluding keratinocyte transcriptome comparison across single‐cell studies. As such, we enriched whole‐tissue RNAseq for keratinocyte‐expressed genes.^19^ As the presence of non‐keratinocyte cells in the whole‐tissue data will likely influence gene expression in those studies, we do not claim that the pathways or functions identified in this current study are unique to keratinocytes, but that they are enriched in keratinocytes. Furthermore, RNAseq analysis of patient samples can only give a snapshot of gene expression. However, these data provide unparalleled depth of gene expression changes in AD skin, allowing holistic evaluation of the transcriptome and the impact of treatment. More single‐cell RNAseq studies, potentially from different disease phases of AD would help our understanding of the immune landscape in AD. Longer term dupilumab transcriptomic studies will allow for future multi‐study analyses to improve understanding of the long‐term effects of this therapy and potential disease resolution. Further work on the functional impacts of DEGs is needed, in particular to understand the temporal role of EGF family of receptors and ligands in acute and chronic AD.

In conclusion, we have demonstrated that a multi‐study transcriptomic approach is valuable for the identification of novel cellular pathways in AD lesions. Our approach identifies a prominent growth factor signature in keratinocytes of moderate‐to‐severe AD patients. There is no current evidence that we know of to suggest that IL‐4 or IL‐13 signalling contributes to EGF family dysregulation in AD lesional keratinocytes. Our study showed an inability of dupilumab to correct enhanced EGF signalling in lesional skin, thus providing evidence to support a lack of an upstream role for signalling through the shared IL‐4 and IL‐13 receptor IL4RA in modulating EGF signalling in keratinocytes. It is likely that this signature is driven by factors which are not normalized by dupilumab. Further investigation of these pathways and their contribution to AD immune pathology may aid the development of novel treatments to interrupt the upstream pro‐inflammatory signals in this common and debilitating skin disease.

## AUTHOR CONTRIBUTIONS

Conceptualization and Writing–original draft preparation: KT and JP; Formal Analysis and Methodology: KT and HG; Investigation and Visualization: KT; Funding Acquisition and Supervision: KT, PA and JP; Writing–Review and Editing: KT, HG, PA and JP.

## CONFLICT OF INTEREST

Dr Kate Timms' salary was funded by the Sanofi Grant awarded to Drs Arkwright and Pennock. Dr Arkwright has acted on Ad hoc Advisory Medical Boards for Sanofi.

## Supporting information


**Figure S1** Pipeline for identification of suitable genomics studies and subsequent data processing and analysis.
**Figure S2** Comparison of in situ keratinocytes, T cells and macrophages/dendritic cells in atopic dermatitis. In the single‐cell RNA‐seq study by He et al,^19^ keratinocyte, T cell and macrophage/dendritic cell clusters were identified based upon their transcriptomes. Here, we compared the expression of markers for (A) keratinocytes, (B) T cells and (C) macrophages/dendritic cells. (D) Plot of first two principal components between the cell types. Kruskal–Wallis test with Dunn's post hoc test. Comparisons are between keratinocytes (A), T cells (B) and macrophages/dendritic cells (C). * = *p* < 0.05, ** = *p* < 0.01, *** = *p* < 0.001, **** = *p* < 0.0001.
**Figure S3** Comparison of atopic dermatitis transcriptomics between different studies. (A) Distribution of gene expression in the 4 studies. (B–C) Correlation matrix of gene expression between studies for all common genes (B) and differently expressed genes (C). All genes expression values are given as log2 lesional vs non‐lesional fold change (FC). Differently expressed genes are considered *p* < 0.05.
**Figure S4** Inflammatory alterations in keratinocyte‐enriched lesional skin. (A) Top significantly enriched inflammatory canonical pathways in keratinocyte‐enriched lesional skin. (B) Hierarchical clustering heatmap of the log2 fold change (lesional vs non‐lesional) in the KELS datasets. Euclidean distance and complete linkage.
**Figure S5** The influence and expression of cytokines and their receptors. (A) Correlations of the KELS DEGs in the current study with publicly available data for keratinocytes treated in vitro with inflammatory mediators^25–29^ predicted to be upstream of DEGs in keratinocyte‐enriched lesional skin. (B) Differential expression of IL4R and IL13RA1 in lesional vs non‐lesional single‐cells (keratinocytes) and tissue. (C) Differential expression of IL4R, IL13RA1 and IL13RA2 in lesional vs non‐lesional keratinocytes, T cells and macrophages/dendritic cells. Statistical analysis was by two‐way ANOVA with multiple comparisons adjustment by Benjamini and Hochberg. *p < 0.05, **p < 0.01, ***p < 0.001, ****p < 0.0001.
**Figure S6** The impact of growth factor signalling on keratinocyte‐enriched lesional skin in atopic dermatitis. (A) Growth factor and hormone pathways significantly enriched in keratinocyte‐enriched lesional skin. (B) Significantly altered growth factors in keratinocyte‐enriched lesional skin. (C–D) Correlation of keratinocyte‐enriched lesional skin with EGF‐treated keratinocytes^36^ at 50% and 100% confluence.
**Figure S7** Ligand–receptor interactions between keratinocytes, T cells and macrophages/dendritic cells in the PDGF/VEGF family. Crosstalk between PDGF/VEGF family ligands (circles) and receptors (squares) between in situ keratinocytes, T cells and macrophages/dendritic cells in atopic dermatitis lesional skin. Colouration is relative expression in lesional skin. Data adapted from He et al.^19^

**Table S1** Genes for which transcripts were present in all five studies.

**Table S2** Differently expressed genes in keratinocyte‐enriched lesional skin.

**Table S3** Functional enrichment analysis of differently expressed genes in keratinocyte‐enriched lesional skin.

**Table S4** Predicted regulators upstream of differently expressed genes in keratinocyte‐enriched lesional skin.

## Data Availability

Datasets related to this article can be found in processed form in Tables [Link], [Link]. All raw data available at the referenced NCBI GEO accession numbers relating to the specific studies referenced in this current study.
