# Supplementary material for: Keratinocyte EGF signalling dominates in atopic dermatitis lesions: A comparative RNAseq analysis
Source: Exp Dermatol. 2022 May 15;31(9):1373–84. doi: 10.1111/exd.14605 (PMC9545602; doi:10.1111/exd.14605)
Supplement: Supplementary file 1 — Figure S1 Pipeline for identification of suitable genomics studies and subsequent data processing and analysis. Figure S2 Comparison of in situ keratinocytes, T cells and macrophages/dendritic cells in atopic dermatitis. In the single‐cell RNA‐seq study by He et al,19 keratinocyte, T cell and macrophage/dendritic cell clusters were identified based upon their transcriptomes. Here, we compared the expression of markers for (A) keratinocytes, (B) T cells and (C) macrophages/dendritic cells. (D) Plot of first two principal components between the cell types. Kruskal–Wallis test with Dunn's post hoc test. Comparisons are between keratinocytes (A), T cells (B) and macrophages/dendritic cells (C). * = p < 0.05, ** = p < 0.01, *** = p < 0.001, **** = p < 0.0001. Figure S3 Comparison of atopic dermatitis transcriptomics between different studies. (A) Distribution of gene expression in the 4 studies. (B–C) Correlation matrix of gene expression between studies for all common genes (B) and differently expressed genes (C). All genes expression values are given as log2 lesional vs non‐lesional fold change (FC). Differently expressed genes are considered p < 0.05. Figure S4 Inflammatory alterations in keratinocyte‐enriched lesional skin. (A) Top significantly enriched inflammatory canonical pathways in keratinocyte‐enriched lesional skin. (B) Hierarchical clustering heatmap of the log2 fold change (lesional vs non‐lesional) in the KELS datasets. Euclidean distance and complete linkage. Figure S5 The influence and expression of cytokines and their receptors. (A) Correlations of the KELS DEGs in the current study with publicly available data for keratinocytes treated in vitro with inflammatory mediators25–29 predicted to be upstream of DEGs in keratinocyte‐enriched lesional skin. (B) Differential expression of IL4R and IL13RA1 in lesional vs non‐lesional single‐cells (keratinocytes) and tissue. (C) Differential expression of IL4R, IL13RA1 and IL13RA2 in lesional vs non‐lesional kera [file EXD-31-1373-s001.docx]

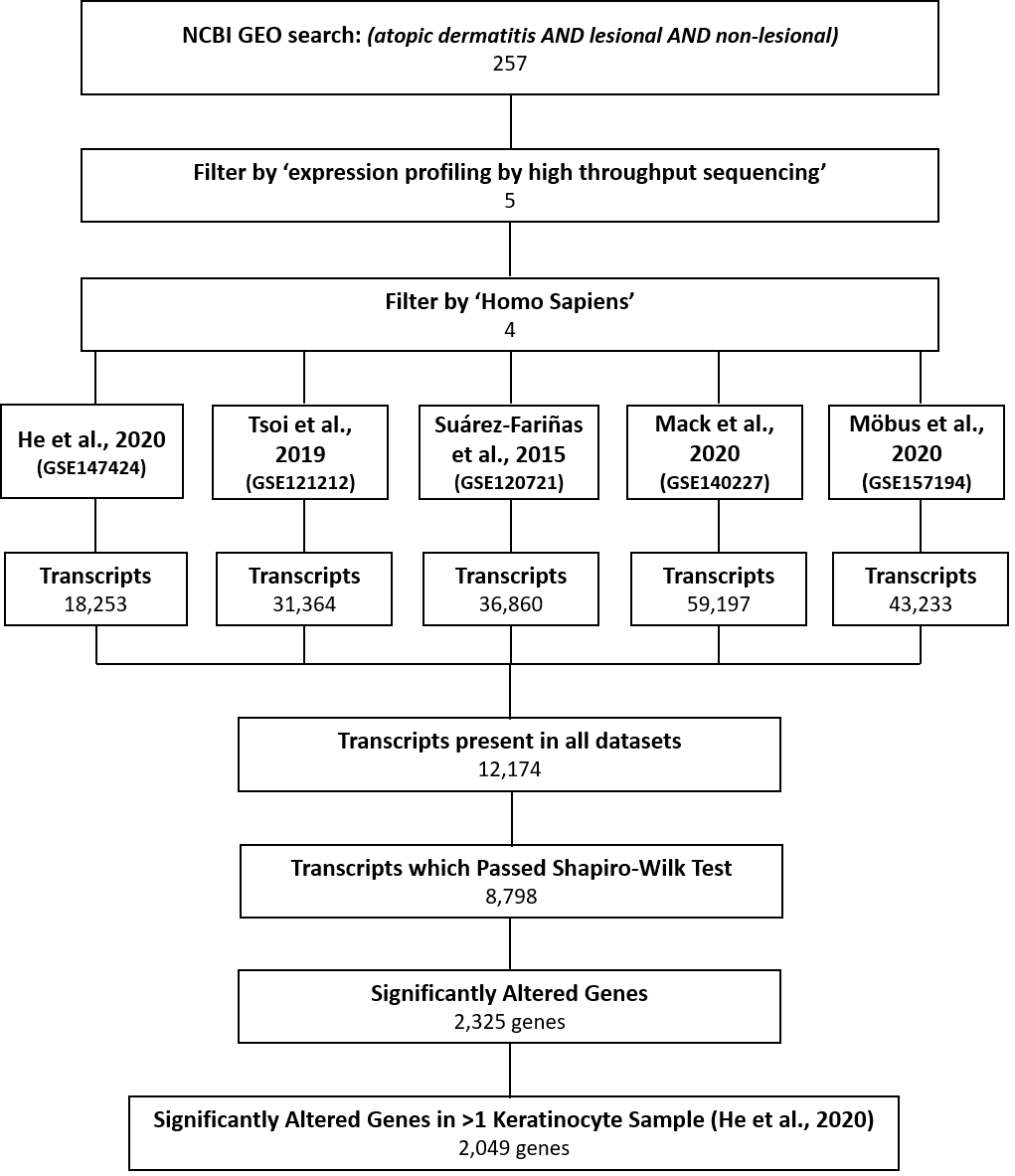


**Supplementary Figure 1: Pipeline for identification of suitable genomics studies and subsequent data processing and analysis.**


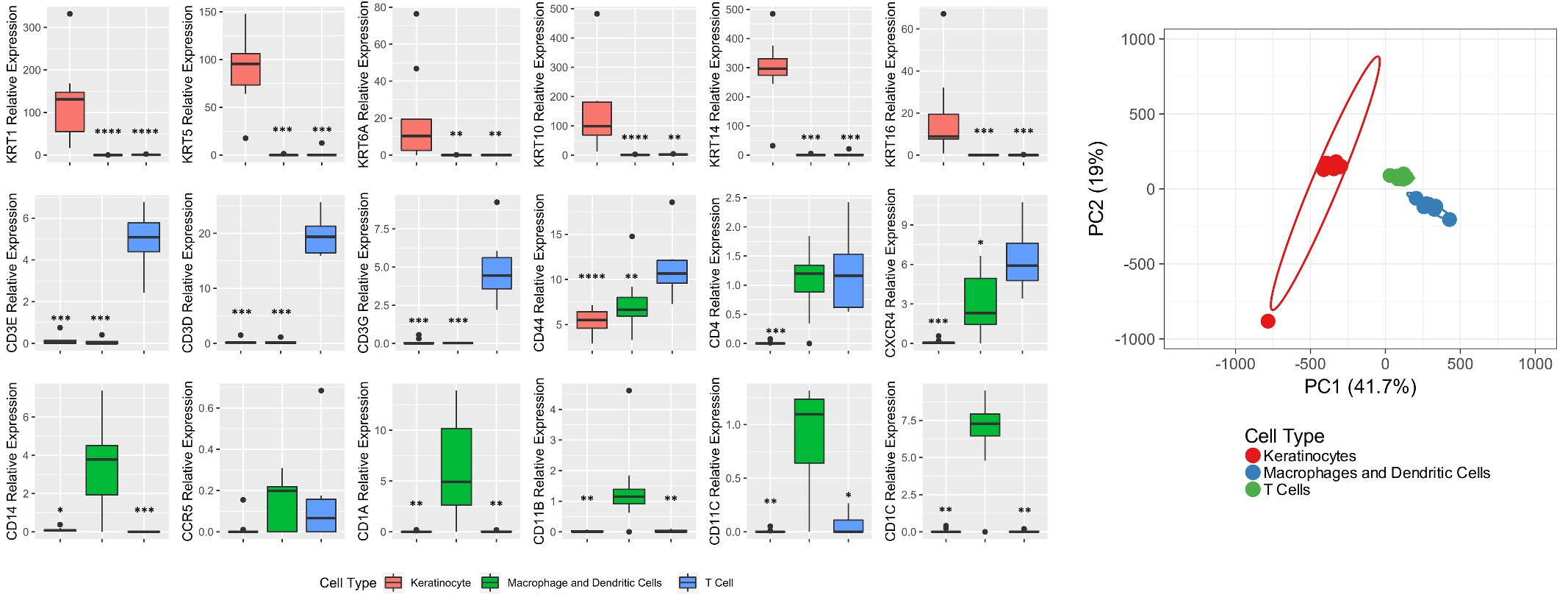


**Supplementary Figure 2: Comparison of *in situ* keratinocytes, T cells and macrophages/dendritic cells in atopic dermatitis.** In the single-cell RNA-seq study by He et al^19^, keratinocyte, T cell and macrophage/dendritic cell clusters were identified based upon their transcriptomes. Here we compared the expression of markers for (**A)** keratinocytes, (**B)** T cells and (**C)** macrophages/dendritic cells. (**D)** Plot of first two principal components between the cell types. Kruskal-Wallis test with Dunn’s post-hoc test. Comparisons are between keratinocytes (A), T cells (B) and macrophages/dendritic cells (C). * = p<0.05, ** = p<0.01, *** = p<0.001, **** = p<0.0001


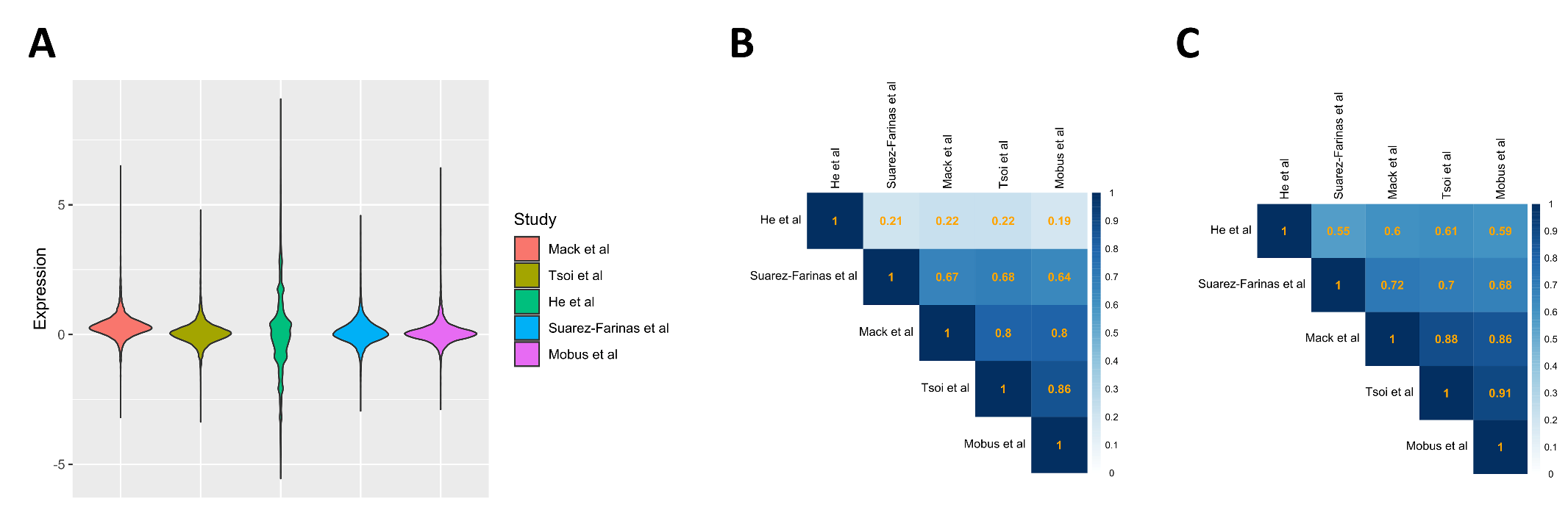


**Supplementary Figure 3: Comparison of atopic dermatitis transcriptomics between different studies.** (A) Distribution of gene expression in the 4 studies. (B-C) Correlation matrix of gene expression between studies for all common genes (B) and differently expressed genes (C). All genes expression values are given as log2 lesional vs non-lesional fold change (FC). Differently expressed genes are considered p<0.05.


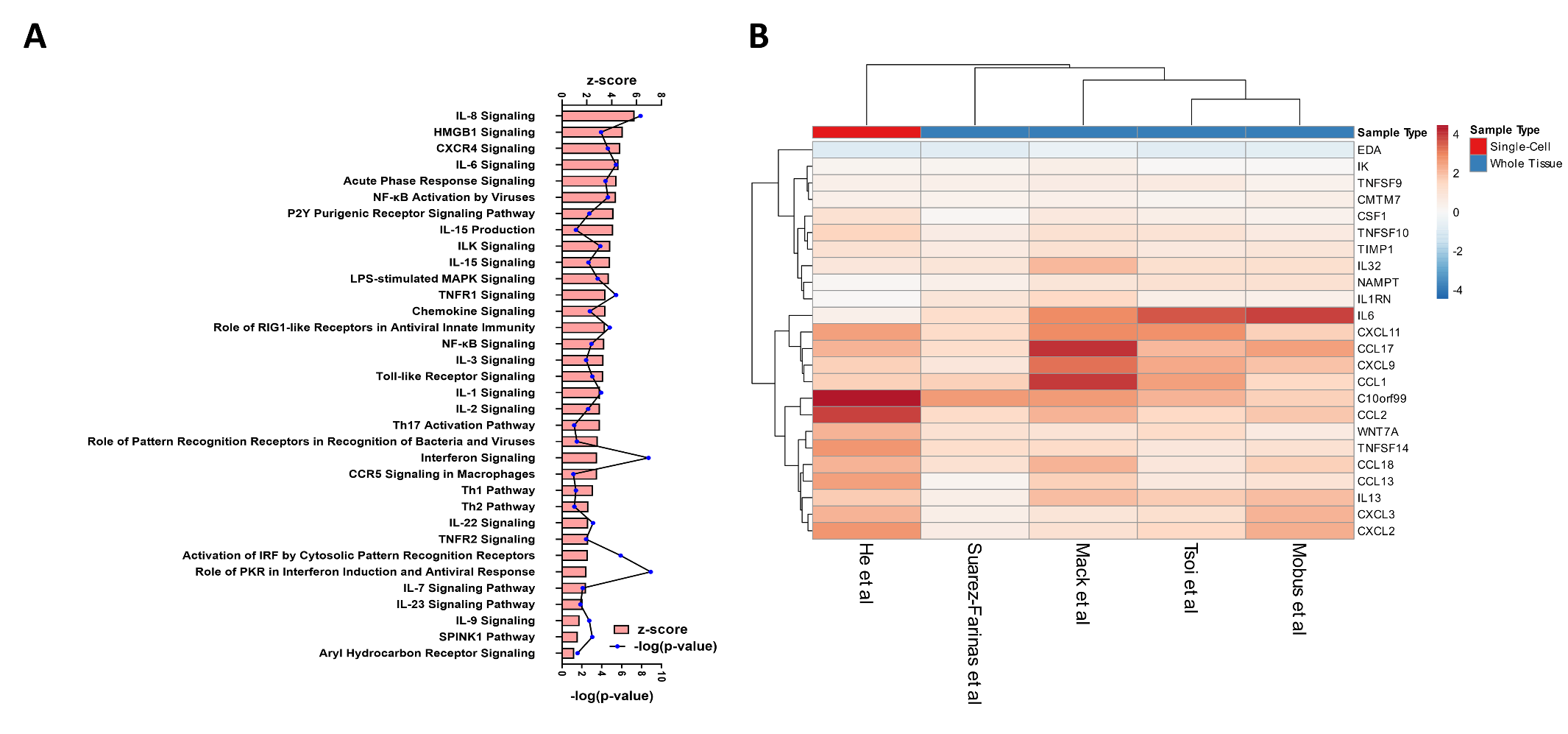


**Supplementary Figure 4: Inflammatory alterations in keratinocyte enriched lesional skin.** (**A**) Top significantly enriched inflammatory canonical pathways in keratinocyte enriched lesional skin. (**B**) Hierarchical clustering heatmap of the log2 fold-change (lesional vs non-lesional) in the KELS datasets. Euclidean distance and complete linkage.


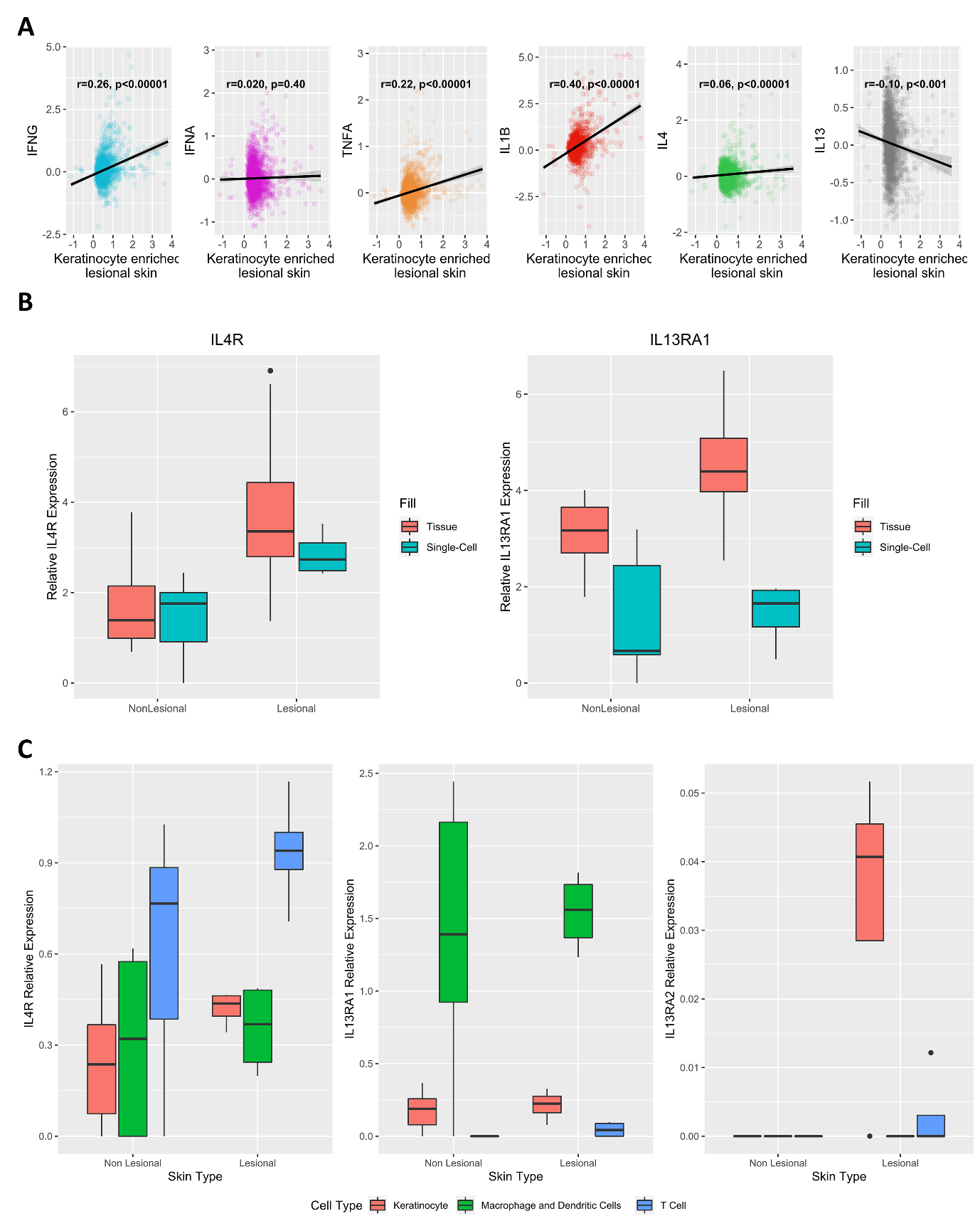


**Supplementary Figure 5:** **The influence and expression of cytokines and their receptors.** (**A**) Correlations of the KELS DEGs in the current study with publicly available data for keratinocytes treated *in vitro* with inflammatory mediators^25-29^ predicted to be upstream of DEGs in keratinocyte enriched lesional skin. (**B**) Differential expression of IL4R and IL13RA1 in lesional vs non-lesional single-cells (keratinocytes) and tissue. (**C**) Differential expression of IL4R, IL13RA1 and IL13RA2 in lesional vs non-lesional keratinocytes, T cells and macrophages/dendritic cells.Statistical analysis was by two-way ANOVA with multiple comparisons adjustment by Benjamini and Hochberg. * = p<0.05, ** = p<0.01, *** = p<0.001, **** = p<0.0001.


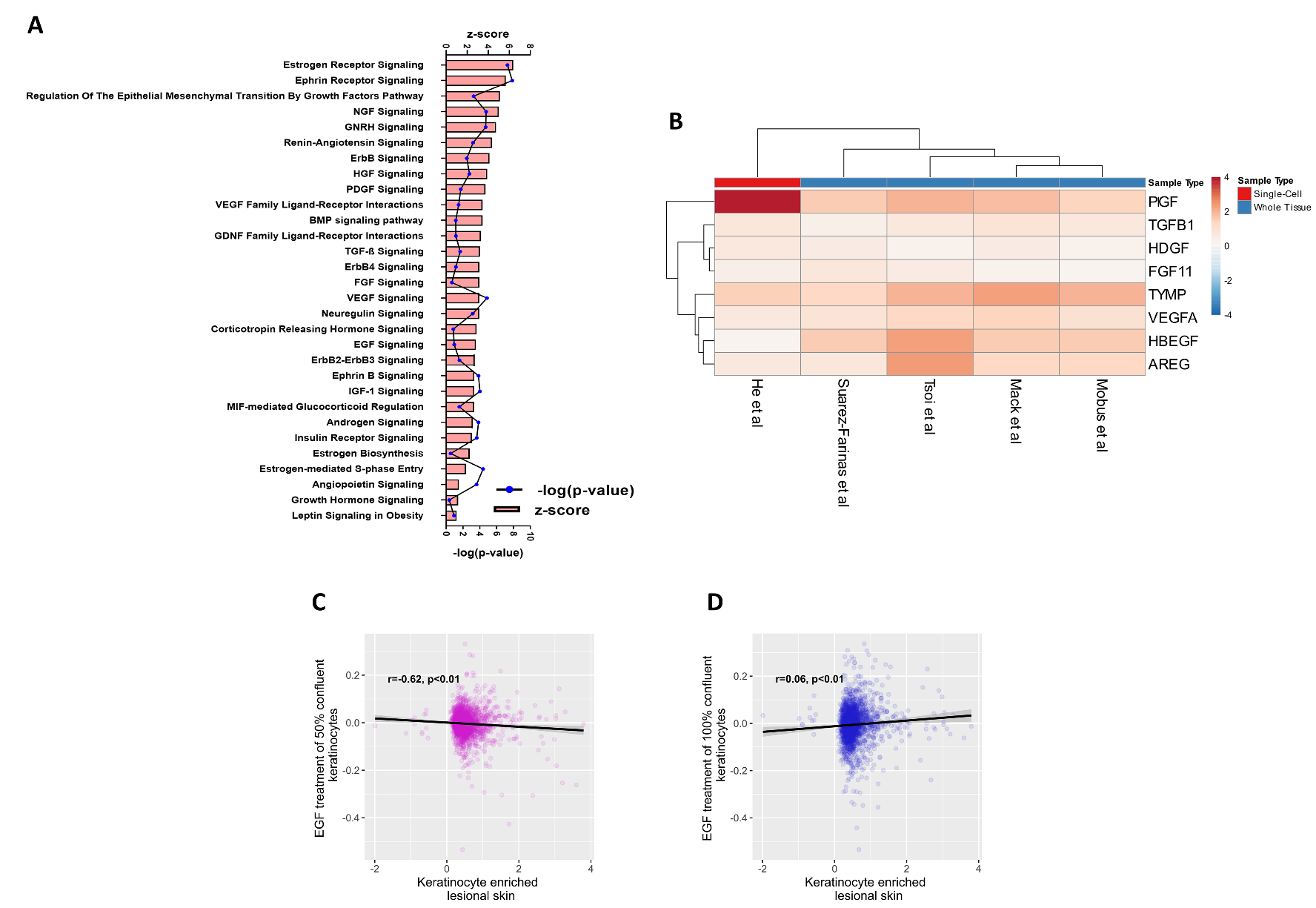


**Supplementary Figure 6: The impact of growth factor signalling on keratinocyte enriched lesional skin in Atopic Dermatitis.** (**A**) Growth factor and hormone pathways significantly enriched in keratinocyte enriched lesional skin. (**B**) Significantly altered growth factors in keratinocyte enriched lesional skin. (**C-D**) Correlation of keratinocyte enriched lesional skin with EGF-treated keratinocytes^36^ at 50% and 100% confluence.


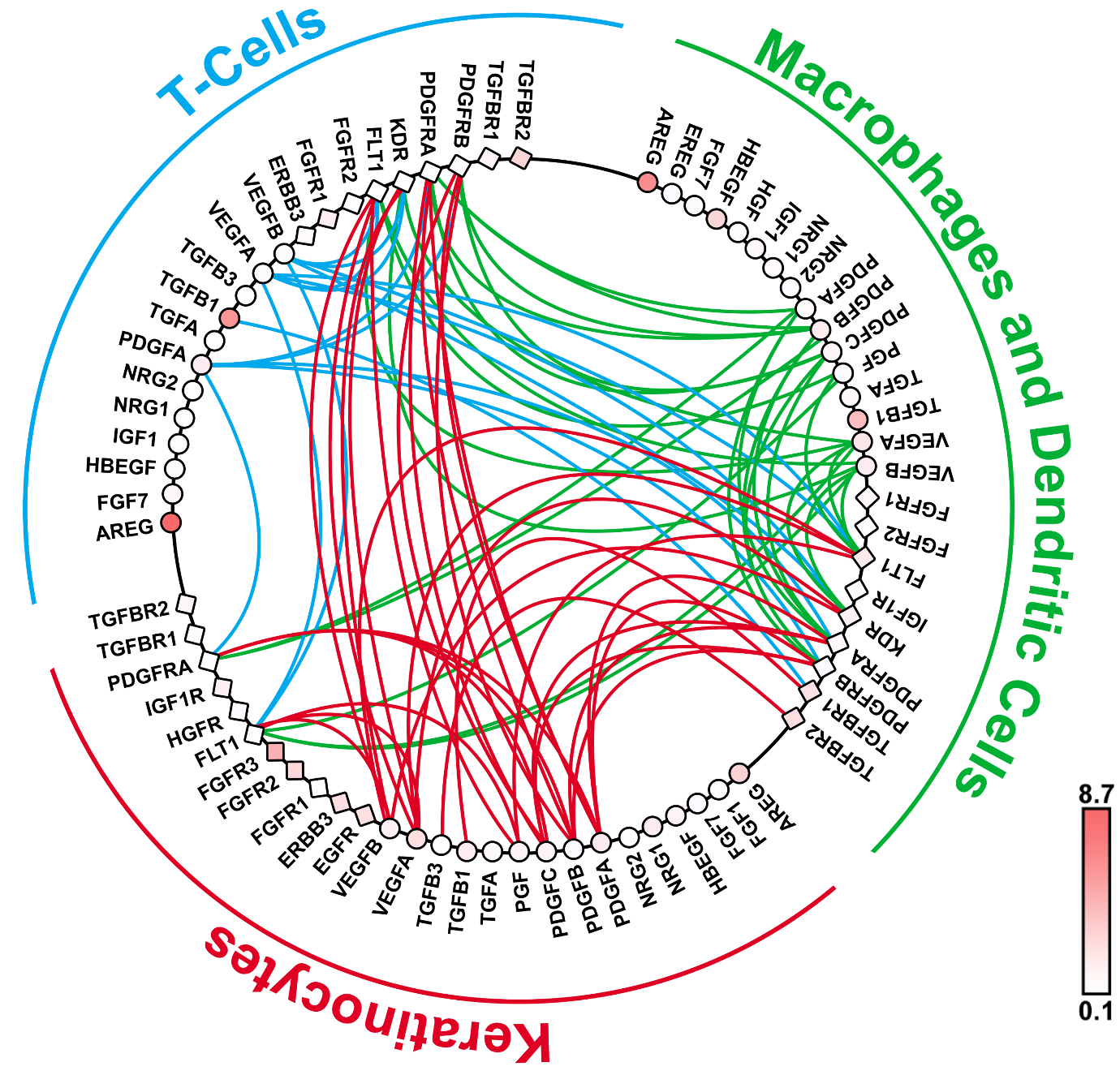


**Supplementary Figure 7: Ligand-receptor interactions between keratinocytes, T cells and macrophages/dendritic cells in the PDGF/VEGF family.** Crosstalk between PDGF/VEGF family ligands (circles) and receptors (squares) between in situ keratinocytes, T-cells and macrophages/dendritic cells in atopic dermatitis lesional skin. Colouration is relative expression in lesional skin. Data adapted from He et al^19^.
